# Nanoparticles Targeting STATs in Cancer Therapy

**DOI:** 10.3390/cells8101158

**Published:** 2019-09-27

**Authors:** Milad Ashrafizadeh, Zahra Ahmadi, Niranjan G. Kotla, Elham Ghasemipour Afshar, Saeed Samarghandian, Ali Mandegary, Abbas Pardakhty, Reza Mohammadinejad, Gautam Sethi

**Affiliations:** 1 Department of Basic Science, Faculty of Veterinary Medicine, University of Tabriz, Tabriz 5166616471, Iran; dvm.milad73@yahoo.com; 2 Department of Basic Science, Shoushtar Branch, Islamic Azad University, Shoushtar 6451741117, Iran; zahra.ahmadi9996@gmail.com; 3 Centre for Research in Medical Devices (CÚRAM), National University of Ireland Galway, Newcastle, Galway H91 W2TY, Ireland; niranjandqa@gmail.com; 4 Neuroscience Research Center, Institute of Neuropharmacology, Kerman University of Medical Sciences, Kerman 7619813159, Iran; elham_gh_afshar@yahoo.com (E.G.A.); alimandegary@yahoo.com (A.M.); drpardakhti@yahoo.com (A.P.); 5 Department of Basic Medical Sciences, Neyshabur University of Medical Sciences, Neyshabur 9318614139, Iran; samarghandians@mums.ac.ir; 6 Pharmaceutics Research Center, Institute of Neuropharmacology, Kerman University of Medical Sciences, Kerman 7616911319, Iran; 7 Department of Pharmacology, Yong Loo Lin School of Medicine, National University of Singapore, Singapore 117600, Singapore

**Keywords:** nanoparticle, drug delivery, STAT3, cancer therapy, bioavailability

## Abstract

Over the past decades, an increase in the incidence rate of cancer has been witnessed. Although many efforts have been made to manage and treat this life threatening condition, it is still one of the leading causes of death worldwide. Therefore, scientists have attempted to target molecular signaling pathways involved in cancer initiation and metastasis. It has been shown that signal transducers and activator of transcription (STAT) contributes to the progression of cancer cells. This important signaling pathway is associated with a number of biological processes including cell cycle, differentiation, proliferation and apoptosis. It appears that dysregulation of the STAT signaling pathway promotes the migration, viability and malignancy of various tumor cells. Hence, there have been many attempts to target the STAT signaling pathway. However, it seems that currently applied therapeutics may not be able to effectively modulate the STAT signaling pathway and suffer from a variety of drawbacks such as low bioavailability and lack of specific tumor targeting. In the present review, we demonstrate how nanocarriers can be successfully applied for encapsulation of STAT modulators in cancer therapy.

## 1. Introduction

As a multidisciplinary field, nanotechnology can be extensively applied in medicine, chemistry and engineering [1,2]. Nanotechnology aims to the development of materials and structures with low size (1–1000 nm) [3]. Over the past decades, significant attention has been directed towards nanotechnology for diagnosis and management of cancer [4]. Clinically, application of a number of strategies such as chemotherapy, radiotherapy and surgery seems to be beneficial in the inhibition of tumorigenesis. However, metastasis and subsequent recurrence are the most challenging problems in cancer therapy [5,6]. Accumulating data demonstrates that there are few major drawbacks associated with conventional cancer therapeutic strategies including the resistance of cancer cells to chemotherapy and radiotherapy, the invasive feature of surgery, unexpected side effects and poor tumor targeting as well as low bioavailability of anti-tumor drugs [7], thereby demanding novel strategies for cancer therapy.

Nanocarriers can be considered as potential candidates in cancer therapy. The low particle size of nanocarriers enables them to effectively penetrate into the blood–brain barrier (BBB) [7]. It appears that application of nanocarriers is associated with enhanced bioavailability of the drug. In fact, nanocarriers can encapsulate the drug to protect it against degradation thus resulting in its enhanced bioavailability for therapeutic application [8]. It is noteworthy that nanoparticles (NPs) provide a minimally invasive-cancer therapy [9] and simultaneously, significantly diminish the chance of resistance and adverse impacts by using a low amount of anti-tumor drug, while the anti-tumor activity is at its highest level [10]. It is possible that mild pH of the tumor microenvironment degrades the drug and more importantly, conventional cancer therapeutic strategies suffer from a lack of specific targeting of cancer cells leading to their toxicity against normal cells. A variety of receptors undergo upregulation in tumor cells and receptor-targeted NPs are of importance in enhancing the delivery of drug into cancer cells [11]. Therefore, based on the high incidence rate of cancer [12], using nanotechnology seems to be a promising approach against this life threatening condition due to its capability in enhancing the anti-tumoral actions of drugs. Currently, various NPs are applied for the delivery of anti-tumor drugs such as solid lipid nanoparticles (SLNs) [13], liposomes [14], niosomes [15], micelles [16], polymeric NPs [17,18,19], carbon nanostructures [20], viral NPs [21], mesoporous silica NPs [22] and gold NPs [23]. Besides, different methods can be used for drug loading. It has been established that various drugs can be predominantly loaded on nanocarriers by encapsulation, as well as covalent or electrostatic binding [24,25,26,27,28].

Cancer is considered as a malignant condition and deregulation of various oncogenic signaling pathways are generally involved in its progression [29]. For example, Wnt signaling pathway is one of the major signaling cascades that can enhance the proliferation and metastasis of cancer cells [30,31,32]. On the contrary, nuclear factor erythroid 2-related factor 2 (Nrf2) can also be targeted to overcome resistance of cancer cells to chemotherapy [33]. These studies demonstrate that diverse oncogenic signaling pathways can be effectively modulated to develop novel strategies for cancer therapy [34,35,36,37]. In the present review, we describe the various ongoing efforts for delivery of anti-tumor drugs primarily targeting oncogenic STAT3 signaling pathway.

## 2. STATs Family: Members and Signaling Pathways

The discovery of signal transducers and activator of transcription (STAT) signaling pathway returns back to 1997, when the scientists have found that STATs are involved in mediation of interferon signaling [38]. A variety of hormones, cytokines and growth factors function as upstream modulators of Janus kinase (JAK)/STAT signaling pathway resulting in regulation of important biological mechanisms such as cell cycle, cell differentiation, cell proliferation and apoptosis [39,40,41,42,43]. Besides, the JAK/STAT signaling pathway is involved in complicated mechanisms such as immune regulation and cancer [44,45]. In mammals, there are four genes encoding JAK1, JAK2, JAK3 and TYK2, and seven genes encoding STAT1, STAT2, STAT3, STAT4, STAT 5A and 5B, and STAT6 [46,47,48]. The expression of JAK3 occurs primarily in hematopoietic cells, while JAK1, JAK2 and TYK2 are ubiquitously expressed [49]. Four major domains are associated with JAKs including N-terminal FERM-domain, SH2-like domain, pseudokinase domain and JH1 domain. It has been demonstrated that FERM and SH2-like domains can contribute to the interaction of JAKs with their receptors [50,51]. On the contrary, STATs effectively affect the transcription of target genes by interaction with DNA regulatory elements (DREs) [52].

Hormones, cytokines and growth factors bind to the receptor leading to the phosphorylation of receptor-associated JAKs. This phosphorylation occurs on the tyrosine (Tyr) residue of JAK that is necessary for stimulation of kinase activity [53]. Importantly, attachment of a ligand to the cell membrane receptor promotes the interaction of receptor-JAK complex to facilitate the phosphorylation of tyrosine residues of cytoplasmic domains of receptors [54], which can than form docking sites for SH2 domain-containing STAT proteins. Then, phosphorylation of Tyr residues within the C terminal domain of receptor-bound STATs occurs resulting in detachment of STATs from receptors and generation of homo- and heterodimers. The STAT proteins accumulate in the cytoplasm and then, translocate into the nucleus where they bind to the members of gamma-activated sites (GASs) and interferon-stimulated response elements (ISREs) [55,56,57,58,59,60]. ISREs are limited to interferon (IFN) signaling, while GASs are present at the promoter of genes including acute-phase proteins [61]. It is noteworthy that STAT3 is capable of transferring from the cytoplasm to nucleus and vice versa, regardless of its phosphorylation status [62].

A number of proteins play a significant role in regulation of the JAK/STAT signaling pathway. These characteristic proteins include the suppressor of cytokine signaling (SCOS), protein tyrosine phosphatases (PTP) and protein inhibitors of activated STATs (PIAS) [63]. SCOS proteins suppress JAK/STAT signaling pathway via A) inhibition of JAK phosphorylation, and B) blocking STAT recruitment [64,65,66]. PIAS proteins prevent the interaction of STAT proteins with DNA. PTP are involved in suppressing JAK proteins [63].

## 3. Role of STATs in Cancer Hallmarks

Importantly, it has been shown that dysregulation of the STAT signaling pathway is associated with development of a number of pathological conditions, particularly cancer. Notably, it seems that STAT1 is considered as a pro-tumorigenic pathway, so that several studies have revealed that the STAT1 signaling pathway significantly enhances the proliferation and malignancy of cancer cells [9,67,68]. However, there are a variety of studies that demonstrate that down-regulation of STAT1 is related to the enhanced invasion and metastasis of tumor cells [69]. Taking these reports into account, dysregulation of STAT1 (upregulation and down-regulation) occurs in tumor cells. It has been shown that interleukin-6 (IL-6) stimulates the malignancy and proliferation of tumor cells. It appears that STAT2 enhances the proliferation of cancer cells by elevating the level of IL-6/STAT3 [70]. A similar story occurs for STAT3, so that various research studies have confirmed that the STAT3 signaling pathway incredibly increases tumor migration, tumor size and tumor malignancy [71,72,73,74,75,76,77]. However, targeting the STAT4 signaling pathway can be considered as a promising strategy in cancer therapy. For example, an upregulation of STAT4 protein can enhance the survival time of patients [78]. Notably, STAT proteins may also act as prognostic signatures in gastric cancer. Moreover, it has been demonstrated that among STAT proteins, STAT4 can determine the prognosis of gastric cancer due to its association with high levels of dendritic cells and CD8+ T cells, whereas STAT3 and STAT6 have minimal prognostic value [79]. Furthermore, miRNA-141-3p inhibits the viability and metastasis of gastric cancer cells through the upregulation of STAT4 [80]. STAT5 and STAT6 contribute in the progression of cancer [41,81,82]. The various members of STAT proteins may also function as upstream modulators of other STAT proteins. STAT5 is an example of this case and it is capable of regulating the expression of STAT3 in tumor cells [83]. Besides, the interaction between STAT proteins may be vital in regulating gene transcription [84]. The STAT signaling pathway can also be involved in the resistance of cancer cells to chemotherapy [85]. For example, accumulating data shows that the RAS signaling pathway may be a key to the malignancy of colorectal cancer (CRC) cells [86,87]. It was found that the interaction between RAS and IFN/STAT signaling pathways [88] can be vital for the induction of the resistance of tumor cells to chemotherapy with trametinib. RAS triggers IFN/STAT signaling pathway by stimulation of STAT1 phosphorylation. Although administration of trametinib is associated with MEK inhibition, the phosphorylation of STAT1 was not found to be affected [89]. IFN/STAT signaling pathway can induce drug resistance in colorectal cancer (CRC) cells via interaction with RAS [89]. Cancer stem cells develop resistance to chemotherapeutic agents by stimulation of the JAK-STAT signaling pathway. Disruption of the JAK-STAT pathway reduces the proliferation and viability capabilities of cancer stem cells [90]. In respect to the potential role of STAT proteins in cancer invasion and metastasis, a number of studies have been performed to elucidate the upstream modulators of STAT signaling pathway. Long non-coding RNA (lncRNA) PART1 is suggested to be involved in enhancing the malignancy of lung cancer cells via induction of JAK-STAT signaling pathway [43]. MicroRNAs (miRs) are short non-coding RNA molecules, which can affect the invasion of cancer cells due to their role in regulation of important biological processes such as cell differentiation, cell proliferation, cell growth and apoptosis [91,92,93]. It appears that miR-15a-3p effectively diminishes the malignancy of liver cancer cells by down-regulation of STAT3 [94]. SOCS plays a significant role in induction of immune system [95]. Moreover, in lung cancer, a reduction in SOCS3 enhances the expression of STAT3 thus causing the progression of cancer cells. MiR-410 down-regulation increases the expression of SOCS3 leading to the decreased level of STAT3 protein and minimized progression of lung cancer cells [96]. Notably, application of STAT3 inhibitor is suggested to be beneficial in the treatment of head and neck cancers [97]. These findings highlight this notion that STAT signaling pathway perturbation is involved in various cancers and targeting this pathway using synthetic or naturally occurring drugs is of importance in cancer therapy. Besides, detecting the mediators of the STAT signaling pathway such as lncRNAs and miRs can be beneficial in genetic manipulation. Based on the complexity and dynamic feature of the STAT signaling pathway, providing an effective modulation of the STAT pathway depends on targeting various signaling molecules involved in regulating this multifunctional pathway.

## 4. STATs Inhibitors

Contemporary therapy is based on targeting the pathways and mechanisms that diseases use. To accomplish this, we should first identify these mechanisms and then create individual molecular drugs that specifically target these pathways. From the theoretical standpoint, targeting one pathway seems very beneficial, but in practice this single therapy is not completely effective and we have not witnessed substantial progress in the eradication of sophisticated pathological disorders, particularly cancer. Besides, using one drug enhances the chance of resistance, so the application of several drugs that affect various molecular pathways diminishes the risk of resistance developing. The targeted therapy of STATs has been advanced due to identification of the unique roles of STATs in various cellular processes. However, over the recent decades, natural and synthetic inhibitors have been developed that can target STAT signaling pathway in various disorders, specifically cancer [98,99,100,101,102,103,104,105,106,107,108,109,110,111,112,113]. Among the STAT proteins, there have been many efforts to detect the inhibitors of STAT3, leading to development of more synthetic and naturally occurring inhibitors of STAT3 compared to other STAT proteins. This may be due to this fact that STAT3 and STAT1 proteins are involved in the progression of several tumor cells [114,115]. It can be concluded that STAT3 inhibitors may negatively affect STAT3 signaling pathway via four major actions [116]: i) Inhibition of SH2 domain or dimerization, ii) influencing upstream mediators of STAT3 such as JAK, iii) suppressing STAT3-DNA domain binding, and iv) endogenous modulators of STAT3. However, there are a variety of difficulties that restrict targeting STAT signaling pathway. For instance, it has been demonstrated that there is a similarity among the structures of STAT proteins, leading to reduced specificity in targeting. Moreover, there is a need for more studies to confirm the safety of these inhibitors in clinical trials.

Furthermore, there have been some attempts to interfere with the transcription of genes. However, these strategies suffer from low specificity and a lack of knowledge about appropriate therapeutic doses [117].

Curcumin is a naturally occurring nutraceutical compound with diverse pharmacological impacts such as antioxidant, anti-inflammatory, anti-diabetic and anti-tumor [118,119,120,121]. It appears that curcumin is capable of targeting different signaling pathways in stimulation of its anti-tumor activity and JAK-STAT pathway is one of them [122,123,124,125]. The induction of apoptotic cell death in H-Ras human mammary epithelial cells is a consequence of direct interaction of curcumin with cysteine (Cys) 259 residue of STAT3. This interaction can lead to the inactivation of STAT3 and subsequently, sensitize tumor cells into apoptotic cell death [126]. Pravastatin is one of the key members of statins with the capability of reducing cholesterol and improving cardiovascular parameters [127]. The administration of pravastatin has been found to be associated with down-regulation of IFN-γ levels and amelioration of atherosclerosis via reducing the expression of STAT1 phosphorylation [128]. It has been demonstrated that pimozide as a neuroleptic drug is capable of targeting STAT proteins [129]. Pimozide can remarkably diminish the phosphorylation level of STAT5 resulting in high cytotoxicity against K562 cells [130]. As an immunosuppressive compound, leflunomide effectively inhibits IgG1 generation by suppressing tyrosine phosphorylation of JAK3 and STAT6 [131]. Niflumic acid has demonstrated great potential in treatment of asthma by modulation of STAT signaling pathway. It seems that IL-13 is vital in induction of asthma through stimulation of chronic inflammation, eosinophilic infiltration, reversible airway narrowing and airway hyperresponsiveness (AHR) [132,133,134,135]. Niflumic acid prevents IL-13-mediated asthma by down-regulation of JAK2 and STAT6 [136]. Cinnamon has a long story in traditional medicine and is extensively used in amelioration of pathological conditions, particularly cancer [137]. The immunomodulatory impact of cinnamon can be attributed to the modulation of STAT proteins, as it suppresses the expression of STAT4 to inhibit the production of IFN-γ [138]. Taking these reports into account, it appears that inhibiting the phosphorylation may be an important strategy for STAT suppression. However, some of them directly bind to the target STAT and suppress its activity. Table 1 and Table 2 summarize the selected pharmacological inhibitors of STAT proteins.

## 5. STATs Gene Silencing by RNA Interference

The introduction of the RNA interference (RNAi) mechanism returns back to two decades ago [163]. This phenomenon has resulted in a great advancement in the investigation of the function of RNAs [12]. At this mechanism, small RNAs containing 18–30 nucleotides are designed to act on long RNAs. This action involves stimulation or inhibition of cleavage at the post-transcriptional level [164]. In respect to the modulatory effect of RNAs on STATs, it seems that regulation of RNAs using RNAi is beneficial in the treatment of pathological disorders associated with dysregulation of STAT proteins [165]. Modulation of STAT3 using RNAi is advantageous in treatment of a laryngeal tumor. An animal model was induced to examine the anti-tumor activity. This animal model included immunocompromised mice in that HepG2 cells were transplanted. Suppressing STAT3 protein remarkably diminished the growth rate of tumors. It appears that STAT3 down-regulation is associated with reduced expression of *Bcl-2*, *cyclin D1* and *survivin* genes leading to the stimulation of apoptotic cell death [166]. A similar observation was noted in pancreatic cancer cells [167], where after suppressing STAT3 expression using STAT3 short hairpin RNA (shRNA) expression vectors, the malignancy and metastasis of pancreatic cancer cells remarkably reduced. Besides, the mRNA expression of matrix metalloproteinase-2 (MMP-2) and the vascular endothelial growth factor (VEGF) underwent down-regulation after STAT3 knockdown, demonstrating the pivotal role of STAT proteins in progression of cancer cells. In spite of much progress in cancer therapy and developing novel drugs targeting various signaling pathways, scientists are not yet able to effectively remedy this life threatening condition. Another study puts emphasis on the potential role of STAT3, STAT5A and STAT5B in the malignancy and invasion of leukemia. In this study, K-562 cells were transfected by anti-STAT3, anti-STAT5A and anti-STAT5B small interfering RNAs (siRNAs). Importantly, the expression of mentioned STAT proteins significantly reduced. It was found that preventing the expression of STAT3, STAT5A and STAT5B is related to the enhanced apoptosis in cancer cells [168]. Finding a new way in treatment of astrocytoma attracts much attention due to the high incident rate of this primary central nervous system tumor. Based on the vital role of STAT3 in the malignancy of tumor cells, inhibition of STAT3 in astrocytoma cells can diminish the mortality resulted from this disorder [169]. STAT3 knockdown promotes the sensitivity of astrocytoma cells into apoptosis.

Furthermore, in respect to the role of STAT3 in inducing the expression of anti-apoptotic factors such as Bcl-xL and survivin, down-regulation of STAT3 is related to the decreased viability and proliferation of cancer cells. However, scientists have faced challenges in the treatment of other brain tumors, particularly glioblastoma. In spite of much effort in the treatment of glioblastoma, it still remains one of the most malignant brain tumors [170]. The capabilities of cells to initiate, progress and recur have led to the high malignancy of these tumor cells [171,172,173,174,175]. Gene manipulation is of importance in reducing the malignancy of glioblastoma cells. Interestingly, inhibition of STAT3 using RNAi can stimulate apoptotic cell death in glioblastoma cells by upregulation of caspase-3 and BAX, and down-regulation of Bcl-2 and cyclin-D. Besides, STAT3 inhibition decreases the CD133+ cell proportion and subsequently, sensitizes cancer cells to apoptosis [176]. On the other hand, one of the difficulties in radio- and chemo-therapy is the resistance of cancer cells. Investigation of molecular signaling pathways and subsequently, regulation of them can be beneficial in enhancing the efficacy of radio- and chemo-therapy. It seems that STAT3 knockdown remarkably elevates the efficacy of radio-therapy in laryngeal carcinoma by reducing the expression of Bcl-2 and VEGF, and enhancing the number of apoptotic cell death [177]. These studies obviously highlight this fact that STAT proteins have vital roles in migration, proliferation and malignancy of cancer cells and modulation of their expression using RNAi interference is a great strategy in combating cancer cells.

## 6. Nano-Technological Approaches for Targeting STATs

### 6.1. Nanoparticles

#### 6.1.1. In Vitro

Based on the statistics reported by American Cancer Society, the efforts for management of cancer should be continued to prevent the high mortality and morbidity associated with this life threatening condition [178]. Cancer cells apply various signaling pathways to ensure their progression. These dynamic and flexible molecular pathways provide a challenge in the treatment of cancer [9,179,180]. On the other hand, although anti-tumor drugs targeting signaling pathways have been introduced in cancer therapy, low bioavailability and lack of targetability diminish the anti-tumor activity of these drugs. To date, NPs have been used for the treatment of various pathological disorders [180] and this capability has been applied in cancer therapy. Hydroxyapatite (HAP) is an important biomaterial with extensive applications in tissue engineering and bone repair [181,182]. HAP has demonstrated great potential in the delivery of DNA and proteins due to its excellent properties such as biocompatibility and porosity [183]. HAP-based NPs can be considered as a promising strategy in the delivery of anti-STAT3 shRNA. HAP NPs effectively deliver anti-STAT3 shRNA to prostate cancer cells leading to the induction of apoptosis and decreased viability of cancer cells. During this transfection, STAT3 down-regulation significantly diminished the expression of Bcl-2, VEGF and cyclin D1. Furthermore, the expression of caspase-3 and BAX underwent upregulation [184]. SLNs are another option in the delivery of small molecule drugs and genetic materials. High biocompatibility and great stability have resulted in application of SLNs for gene delivery [185]. Loading a STAT3 inhibitor on SLNs is of importance in combating lung cancer cells. SLNs protected genetic materials against DNasel and serum-mediated degradation. Encapsulation of DNA by SLNs preserved its supercoiled and circular formation. STAT3 inhibitor-loaded SLNs significantly sensitized lung cancer cells to cisplatin-mediated apoptosis (Table 3) [186]. In respect to the potential role of STAT3 in enhancing the malignancy of cancer cells [187], this signaling pathway has obtained much attention in triple negative breast cancer (TNBC) therapy and a number of drugs approved by the Food and Drug Administration (FDA) such as niclosamide have been used in treatment of TNBC as inhibitors of STAT3 [188]. In accordance to the efficacy of SLNs in the delivery of STAT inhibitors, loading a STAT3 inhibitor on SLNs remarkably decreases the viability of cancer cells by stimulation of apoptosis via down-regulation of STAT3 phosphorylation [13]. SLNs have been applied in treatment of ovarian cancer due to their potential in delivery of STAT3 siRNA and consequently, stimulation of apoptotic cell death through down-regulation of Bcl-2 and survivin [189].

Accumulating data demonstrates that SHP-1 may be able to modulate stemness and the epithelial-to-mesenchymal transition (EMT) of tumor cells by targeting the JAK2/STAT3 signaling pathway [190,191,192]. Therefore, NP-mediated SHP-1 regulation is of interest in cancer therapy. ZnAs@SiO_2_ NPs use the same strategy in reducing hepatocellular carcinoma malignancy. It seems that application of ZnAs@SiO_2_ NPs significantly diminishes the expression of stemness markers such as CD133, Sox-2 and Oct-4. Besides, these NPs are capable of induction of apoptotic cell death and reducing the metastasis and migration of hepatocellular carcinoma cells by EMT inhibition. These anti-tumor activities arise as a result of disruption in the SHP-1/JAK2/STAT3 signaling pathway [193]. Receptor-targeted delivery enhances the capability of NPs in decreasing the viability of cancer cells. It has been demonstrated that CD38 has a minimal expression in normal cells, while its overexpression occurs in multiple myeloma (MM) cells [194]. There have been efforts to target CD38 at MM cells and daratumumab has been used for this purpose [195,196,197]. Moreover, anti-CD38-decorated NPs carrying the STAT3 inhibitor have been reported to have high cellular uptake with great anti-tumor activity [198].

Poly(lactic-co-glycolic acid) (PLGA) has a variety of excellent properties such as biocompatibility and biodegradability. FDA has approved the application of PLGA for human uses. PLGA NPs have a size similar to pathogens leading to their phagocytosis by dendritic cells (DCs). This feature has resulted in application of PLGA NPs for delivery of drugs into DCs [199,200,201,202,203,204]. It appears that PLGA provides a suitable platform for conjugation of JSI-124, as a STAT3 inhibitor. JSI-124 PLGA NPs have great anti-tumor activity against B16 melanoma cells by reducing the expression of STAT3 in DCs and enhancing the function of DCs in terms of promoting the production of T cells leading to the cancer immunotherapy [205]. The capability of PLGA NPs in releasing drugs in a sustained-released behavior is of importance in co-delivery of paclitaxel, a chemotherapeutic agent and STAT siRNA to sensitize lung cancer cells to apoptotic cell death [206]. Taking everything into account, in respect to the ability of PLGA NPs in delivery, anti-STAT3-loaded PLGA NPs can be considered as promising agents in cancer immunotherapy by targeting DCs [207]. It has been demonstrated that STAT3-siRNA-loaded NPs have high cellular uptake by tumor cells leading to their high efficacy in reducing the malignancy of cancer cells. It appears that clathrin-mediated endocytosis participates in cellular uptake of STAT3-siRNA-loaded NPs by melanoma cells [208].

#### 6.1.2. In Vivo

Melanoma is one of the malignant skin cancers that proliferation of pigment producing melanocytes occurs in the epidermis. Surgery and chemotherapy are considered as current strategies in melanoma therapy [209,210,211]. However, one of the problems associated with chemotherapy is the resistance of tumor cells [29]. Using gene therapy enhances the anti-tumor activity of chemotherapeutic agents. Co-delivery of imatinib and anti-STAT3 siRNA (non-invasive topical iontophoretic administration) using gold NPs is related to a remarkable decrease in tumor volume and tumor weight in melanoma tumor bearing mice, showing the efficacy of gold NPs in treatment of melanoma by inhibition of STAT3 [212]. Erlotinib (ELTN) is extensively used in chemotherapy with the capability of targeting epidermal growth factor receptor (EGFR) gene. However, resistance of cancer cells challenges the potential of this agent in chemotherapy [213,214]. Fedratinib (FDTN) is a small molecule known as the JAK2 inhibitor and is applied in the treatment of myelofibrosis [215]. Co-administration of ELTN and FDTN using biodegradable NPs leads to the satisfactory results in ELTN-resistance non-small cell-lung cancer (NSCLC) cells. The biodegradable NPs had great stability and effectively released drug at mild acidic pH of the tumor microenvironment. Loading a combination of ELTN and FDTN on NPs not only enhances the anti-tumor activity by inhibition of the JAK2/STAT3 signaling pathway, but also diminishes the systemic adverse effects [216]. As it was mentioned, HAP has human applications due to its high biocompatibility. Besides, it seems that HAP has anti-tumor activity making its appropriate for cancer therapy [217,218,219,220,221,222,223]. HAP NPs are capable of inhibiting the progression and invasion of prostate tumor cells in mouse model by reducing the expression of STAT3 resulting in down-regulation of Bcl-2, VEGF and cyclin D1 [224]. A newly developed nanocarrier for the delivery of siRNA should be capable of protection of siRNA against degradation, promoting siRNA potency and simultaneously, improving the biodistribution and pharmacokinetics [224]. Polymeric NPs have demonstrated great potential in this field and polyethyleneimine is among them [225,226,227,228]. Loading STAT3 siRNA on lipid-substituted PEI is associated with decreased viability and proliferation of tumor cells by upregulation of caspase-3 and IL-6, and down-regulation of STAT3 and VEGF [229].

5,2^/^-4^/^-trihydroxy-6,7,5^/^-trimethoxyflavone (TTF1) is a naturally occurring compound exclusively found in *Sorbaria sorbifolia* (SS) [230,231]. TTF1 has great pharmacological effects such as anti-tumor activity. However, low bioavailability and biodegradation restrict the therapeutic activities of TTF1 [232]. TTF1-loaded NPs are able to remarkably suppress angiogenesis and metastasis of human hepatoma cancer cells by down-regulation of STAT3. It appears that decreased invasion of cancer cells is a consequence of MMP-2 and MMP-9 down-regulation. Besides, anti-angiogenic effect of TTF1-NPs is mediated by reducing the expression of VEGF [233]. BBB is considered as one of the most challenging problems in penetration of drugs into brain. PEI-PLGA NPs solve this problem by enhancing the crossing of STAT3 siRNA through BBB [234].

### 6.2. Liposomes

#### 6.2.1. In Vivo

It seems that liposomes are potential candidates in the treatment of skin cancer. This notion emanates from the capability in crossing over the stratum corneum layer of skin [235]. It has been demonstrated that edge activators (transferosomes)- or ethanol (ethosomes)-based liposomes are able to deeply penetrate into the skin [236]. Besides, physical techniques such as iontophoresis have enhanced the penetration potential of liposomes into the skin [237,238,239,240]. Therefore, liposomes can serve as promising candidates for delivery of STAT proteins in the treatment of skin cancers [241]. It appears that curcumin- and STAT3 siRNA-loaded liposomes significantly down-regulate the expression of STAT3 protein leading to the inhibition of tumor invasion and a remarkable reduction in tumor weight and tumor volume [242].

#### 6.2.2. In Vitro

Targeting tumor-associated macrophages (TAMs) is of importance in cancer therapy due to the potential role of TAMs in the tumor microenvironment and enhancing the malignancy, invasion, angiogenesis and resistance of cancer cells [243,244]. It is held that enhanced TAM-infiltration is associated with a decrease in survival time of patients with cancer [245,246,247]. Notably, disruption in the STAT3 signaling pathway effectively promotes anti-tumor immunity by enhancing the production of TNF-α and stimulation of M1-like reprogramming of macrophages [248,249,250,251,252]. Hence, providing STAT3 modulation in macrophages is of interest in improving anti-tumor immunity. CD163-targeted crosolic acid-containing liposomes prevent the expression of STAT3 in macrophages, resulting in enhanced anti-tumor immunity by increasing TNF-α, IFN-γ, IL-12 and IL-2 levels, and decreasing the IL-10 level [245]. Similar to in vivo findings, co-delivery of curcumin and STAT3 by deformable cationic liposomes is associated with cell growth inhibition and apoptosis induction. It is held that clathrin-induced endocytosis mediates the penetration of liposomes into skin [241].

### 6.3. Micelles

#### In Vitro and In Vivo

**Figure 1:**
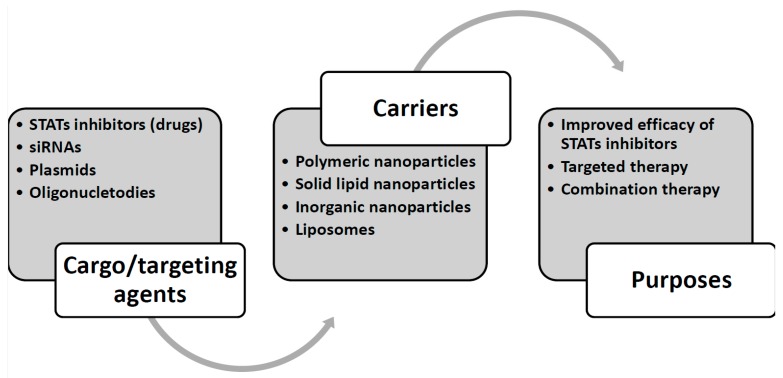
Application of nanoparticles in targeting STATs.

Micelles were first introduced in 1984 for the delivery of drugs [253,254]. Micelles are able to remarkably improve the bioavailability and anti-tumor activity of drugs [255,256]. It seems that polymeric micelles have higher permeability and retention effect compared to the conventional micellar nanocarriers [257,258] making them appropriate for drug delivery. There are two studies that have investigated the efficiency of micellar NPs in the delivery of STAT inhibitors in melanoma cells both in vitro and in vivo. It was found that administration of STAT3 inhibitor-loaded polymeric micelles results in apoptotic cell death in melanoma cells and down-regulates VEGF expression. Besides, these nanocarriers have greater biocompatibility and improve anti-tumor immunity by enhancing DC-mediated IL-12 production [259,260]. The potential application of nanoparticles in targeting STATs is summarized in Figure 1.

## 7. Conclusion and Future Trends

In respect to the vital role of STAT proteins in various important biological processes including cell cycle, differentiation, apoptosis and cell proliferation, any impairment in the STAT signaling pathway is associated with the development of pathological conditions, particularly cancer. As a consequence, targeting the STAT signaling pathway has demonstrated a great potential in cancer therapy. On the other hand, there have been some difficulties in the delivery of drugs that target the STAT signaling pathway. Therefore, it seems that application of nanocarriers for loading STAT modulators may be important in terms of releasing drug into the tumor site and inhibition of resistance of cancer cells by loading the optimum amount of drug. Until now, various nanoparticles have been designed for targeting the STAT signaling pathway, especially STAT3, which include gold nanoparticles, hydroapatite nanocarriers, PLGA nanoparticles, micelles, solid lipid nanoparticles, liposomes and microbubbles. These nanocarriers have been applied in various cancers both in vitro and in vivo, and exhibited high potentiality in reducing the viability, migration and malignancy of tumor cells by regulating the expression of STAT proteins. However, more studies are needed to elucidate the efficacy of nanoparticles in targeting the STAT signaling pathway for cancer therapy. Although huge emphasis has been put on the capabilities and benefits of using NPs in delivery of STAT to cancer cells, it has been reported that only 0.7% of administered NPs are found to be delivered to the tumor site, thereby challenging the potential role of NPs in drug delivery [271]. This issue needs to be carefully addressed in future studies.

## Figures and Tables

**Table 1:** Signal transducers and activator of transcription (STAT) inhibitors except STAT3 inhibitors.

Drug	Molecular Formula	Target	Effect	Animal Model/Cell Line	Refs
---	2-(3′,4′,5′-trimethoxybenzoyl)-3-iodoacetamido-6-methoxy benzo[b]furan derivative 1	STAT5	Inhibition of STAT5 phosphorylation	K562 cells	[139]
---	N’-(4-Oxo-4 H-chromen-3-yl)methylene) nicotinohydrazide	STAT5	Inhibition of STAT5 phosphorylation	Chronic myeloid leukemia (CML) cells	[140]
SEL120-34A	C_15_H_19_Br_2_C_l_N_4_	STAT1, STAT5	Inhibition of STAT1 S727 and STAT5 S726 phosphorylation	Acute myeloid leukemia (AML) cells	[141]
R763	---	STAT5	Inhibition of STAT5 phosphorylation	Neoplastic mast cell	[142]
Pravastatin	C_23_H_36_O_7_	STAT1	Prevention of STAT1 expression	Mice	[128]
Pimozide	C_28_H_29_F_2_N_3_O	STAT5	Inhibition of STAT5 phosphorylation	K562 cells, peripheral T-cell lymphoma	[130,143]
Leflunomide	C_12_H_9_F_3_N_2_O_2_	STAT6	Inhibition of tyrosine phosphorylation of STAT6	B cells	[131]
Niflumic acid	C_13_H_9_F_3_N_2_O_2_	JAK2, STAT6	Blockade of STAT6 phosphorylation	Mouse	[136]
Cinnamon	C_36_H_32_O_19_	STAT4	Blockade of STAT4 phosphorylation	Mice	[138]
Atiprimod	C_22_H_44_N_2_	STAT5STAT3	Inhibition of phosphorylation	AML cells	[144]

**Table 2:** Natural STAT3 inhibitors.

Drug	Molecular Formula	Effect	Animal Model/Cell Line	Refs
Silibinin	C_25_H_22_O_10_	Blocking pathways of STAT3 activation	Endometrial carcinoma cells	[145]
Quercetin	C_15_H_10_O_7_	Inhibiting STAT3 signaling pathways	Lymphoma cells	[146]
Berberine	C_20_H_18_NO_4+_	Decrease of STAT3 phosphorylation	Keratinocytes	[147]
Resveratrol	C_14_H_12_O_3_	Inhibition of STAT3	Rat	[148]
Triterpenes from *Helicteres angustifolia*	---	Inhibition of STAT3 phosphorylation	HT-29 colorectal cancer cells	[149]
Butein	C_15_H_12_O_5_	Inhibition of STAT3 expression	Multiple myeloma cells	[150]
Caffeic acid	C_9_H_8_O_4_	Inhibition of activity of STAT3 Inhibition of JAK/STAT3 signaling pathway	Mouse, Human renal carcinoma cells	[151,152]
Capsaicin	C_18_H_27_NO_3_	Inhibition of STAT3	Human multiple myeloma cells	[153]
Celastrol	C_29_H_38_O_4_	Inhibition of STAT3 phosphorylation	Human hepatocellular carcinoma	[154]
Cucurbitacin	C_32_H_48_O_8_	Inhibition of STAT3 activation	AML cells	[155]
Diosgenin	C_27_H_42_O_3_	Inhibition of STAT3 phosphorylation	Human hepatocellular carcinoma cells	[156]
Guggulsterone	C_21_H_28_O_2_	Inhibition of STAT3 phosphorylation	Tumor cells	[157]
Honokiol	C_18_H_18_O_2_	Modulation of STAT3 activation	Breast cancer cells	[158]
Avicin D	C_98_H_155_NO_46_	Inhibition of STAT3 phosphorylation	U266 cells, myeloma cell lines	[159]
Piceatannol	C_14_H_12_O_4_	Reduction of P-STAT3 expression	Mouse	[160]
Withaferin A analogues	---	Inhibition of STAT3 phosphorylation	Breast cancer cell line	[161]
Emodin	C_15_H_10_O_5_	Inhibition of STAT3 phosphorylation	Hepatocellular carcinoma cell lines	[162]

**Table 3:** Potential use of nanocarriers for delivery of STAT inhibitors.

Nano-carriers	Agent	In vitro/In vivo	Cell Line/Animal Model	Major Outcomes	Refs
Gold nanoparticle	STAT3 siRNA and imatinib	In vitro and in vivo	B16F10 (melanoma cells) and tumor bearing C57BL/6 mice	In vitro: Inhibition of tumor growth and decreased expression of STAT3 In vivo: decreased weight and volume of tumor, reduced expression of STAT3	[212]
Hydroxyapatite nanoparticles	Plasmid-based STAT3 siRNA	In vivo	Mouse prostate cancer cells	The downregulation of STAT3 downstream genes such as Bcl-2, VEGF and cyclin D1, and consequently, increased level of apoptosis in cancer cells	[231]
PLGA nanoparticles	siRNA polyplexes	In vitro	DCs	Downregulation of STAT3 expression and increased level of maturation and functionality in DCs	[207]
Micelle	STAT3 siRNA	In vivo	Mice with tumor-associated DCs (TADCs)	Downregulation of STAT3 and stimulation of maturation and activation in TADCs	[261]
Solid lipid nanoparticle	STAT3 decoy oligodeoxynucleotides	In vitro	Human ovarian cancer cell lines A2780 and SKOV3	Inhibition of STAT3 pathway, stimulation of cell death via increased expression of Bax, Beclin-1, caspase-3 and LC3-II, and prevention of invasion via upregulation of E-cadherin and downregulation of Snail and MMP-9	[189]
PEI-PLGA-FITC nanoparticles	siRNA targeting STAT3	In vitro and in vivo	A549 cells and Balb/c mice	In vitro: Reduced rate of viability in A549 cells. In vivo: Upregulation of caspase-3 and downregulation of IL-6 in mice	[234]
Liposome	shRNA against STAT3	In vitro	Ovarian cancer cell lines A2780CP and A2780ss	Increased level of apoptosis and inhibition of cell proliferation	[262]
Poly (D,L-lactic-co-glycolic-acid) nanoparticle	JSI-124 (STAT3 inhibitor)	In vitro	DCs	Improved function of DCs and increased level of T cell proliferation	[205]
Ultrasound-targeted microbubble destruction	Transcription factor decoy of STAT3	In vivo	Squamous cell tumors	Downregulation of STAT3 and inhibition of tumor growth	[263]
Deformable cationic liposomes	Curcumin and STAT3 siRNA	In vitro	Human epidermoid (A431) cancer cells	Inhibition of cancer cell growth and stimulation of apoptosis	[241]
Lipid-substituted polyethylenimine	STAT3 siRNA	In vitro	Murine B16.F10 melanoma cells	Remarkable inhibition of STAT3 expression and induction of apoptosis	[229]
Inorganic kernel-supported asymmetric hybrid vesicles	STAT3-decoy oligonucleotide	In vivo	Nude mice bearing BT474R breast cancer xenograft	Significant inhibition of tumor growth and prevention of trastuzumab resistance	[264]
Self-Associating Poly(ethylene oxide)-block-poly(α-carboxyl-ε-caprolactone) Drug Conjugates	JSI-124 (STAT3 inhibitor)	In vitro	B16F10 melanoma cells and tumor exposed bone marrow derived dendritic cells	Inhibition of STAT3 and great anti-tumor activity	[265]
E-selectin thioaptamer-conjugated multistage vector	siRNA	In vivo	Mice bearing metastatic breast cancer and murine xenograft models of human MDA-MB-231 breast tumor	Downregulation of STAT3 as much as 48.7% in cancer cells inside bone marrow, and increased rate of survival in mice	[266]
Lipid-substituted polyethylenimine	siRNA polyplexes	In vitro	Wild-type MDA-MB-435 breast cancer cells	Downregulation of STAT3 and decreasedviability of cells	[267]
Polymeric nanoparticles	STAT6	In vitro and in vivo	HeLa cells and tumor bearing mice	In vitro: knockdown of IFN-γR2 and stimulation of cell death in HeLa human epithelial cells In vivo: decreased volume of tumor and increased rate of survival	[268]
Gold nanoparticles	STAT3 siRNA	In vitro	B16F10 murine melanoma cells	Remarkable inhibition of cancer cell growth	[208]
Lipid nanoparticle	RNAi-mediating plasmid DNA	In vitro	Chemoresistant Calu1 cells	Downregulation of STAT3 and resensitize Calu human lung cancer cells to chemotherapy (cisplatin)	[186]
PLGA nanoparticles	JSI-124 (STAT3 inhibitor)	In vivo	C57BL/b male mice	Great anti-tumor impact	[269]
Dissolving microneedles	STAT3 siRNA	In vivo	Female C57BL/b mice	Great gene silencing and inhibition of tumor cell growth	[270]

## Abbreviations

BBB	blood-brain barrier
NPs	nanoparticles
STAT	signal transducers and activator of transcription
DRE	DNA regulatory elements
Tyr	tyrosine
GAS	gamma-activated sites
ISREs	interferon-stimulated response elements
IFN	interferon
SCOS	suppressor of cytokine signaling
PTP	protein tyrosine phosphatase
PIAS	protein inhibitors of activated STATs
IL	interleukin
CRC	colorectal cancer
lncRNA	long non-coding RNA
miR	microRNA
Cys	cysteine
AHR	airway hyper responsiveness
RNAi	RNA interference
MMP	matrix metalloproteinase
VEGF	vascular endothelial growth factor
SLNs	solid lipid NPs
HAP	hydroxyapatite
TNBC	triple negative breast cancer
Niclo	niclosamide
FDA	Food and Drug Administration
EMT	epithelial-to-mesenchymal transition
MM	multiple myeloma
DCs	dendritic cells
ELTN	erlotinib
EGFR	epidermal growth factor receptor
FDTN	fedratinib
NSCLC	non-small cell-lung cancer
PEI	polyethylenimine
TTF1	trimethoxyflavone
SS	Sorbaria sorbifolia
TAM	tumor-associated macrophage
TADCs	tumor-associated CDs
JAK	Janus kinase
